# Operations research for resource planning and -use in radiotherapy: a literature review

**DOI:** 10.1186/s12911-016-0390-4

**Published:** 2016-11-25

**Authors:** Bruno Vieira, Erwin W. Hans, Corine van Vliet-Vroegindeweij, Jeroen van de Kamer, Wim van Harten

**Affiliations:** 1 Department of Radiation Oncology, Netherlands Cancer Institute - Antoni van Leeuwenhoek Hospital, Amsterdam, The Netherlands; 2 Center for Healthcare Operations Improvement and Research (CHOIR), University of Twente, Enschede, The Netherlands; 3 Department Industrial Engineering and Business Information Systems, Faculty of Behavioural Management and Social Sciences, University of Twente, Enschede, The Netherlands; 4 Department of Health Technology and Services Research, Faculty of Behavioural Management and Social Sciences, University of Twente, PO Box 217, 7500 AE Enschede, The Netherlands; 5 Rijnstate General Hospital, Arnhem, The Netherlands

**Keywords:** Operations research, Radiotherapy, Literature review, Resource planning, Logistics optimization, Operations improvement

## Abstract

**Background:**

The delivery of radiotherapy (RT) involves the use of rather expensive resources and multi-disciplinary staff. As the number of cancer patients receiving RT increases, timely delivery becomes increasingly difficult due to the complexities related to, among others, variable patient inflow, complex patient routing, and the joint planning of multiple resources. Operations research (OR) methods have been successfully applied to solve many logistics problems through the development of advanced analytical models for improved decision making. This paper presents the state of the art in the application of OR methods for logistics optimization in RT, at various managerial levels.

**Methods:**

A literature search was performed in six databases covering several disciplines, from the medical to the technical field. Papers included in the review were published in peer-reviewed journals from 2000 to 2015. Data extraction includes the subject of research, the OR methods used in the study, the extent of implementation according to a six-stage model and the (potential) impact of the results in practice.

**Results:**

From the 33 papers included in the review, 18 addressed problems related to patient scheduling (of which 12 focus on scheduling patients on linear accelerators), 8 focus on strategic decision making, 5 on resource capacity planning, and 2 on patient prioritization. Although calculating promising results, none of the papers reported a full implementation of the model with at least a thorough pre-post performance evaluation, indicating that, apart from possible reporting bias, implementation rates of OR models in RT are probably low.

**Conclusions:**

The literature on OR applications in RT covers a wide range of approaches from strategic capacity management to operational scheduling levels, and shows that considerable benefits in terms of both waiting times and resource utilization are likely to be achieved. Various fields can be further developed, for instance optimizing the coordination between the available capacity of different imaging devices or developing scheduling models that consider the RT chain of operations as a whole rather than the treatment machines alone.

**Electronic supplementary material:**

The online version of this article (doi:10.1186/s12911-016-0390-4) contains supplementary material, which is available to authorized users.

## Background

Due to the growing numbers of cancer patients, demand for RT has been continuously increasing [1]. According to Delaney et al. [2, 3], the optimal rate for the use of RT in some part of the treatment in cancer care should be around 50%, although this figure has not yet been achieved in practice [4]. In addition, RT has proven to be at least as cost-effective as both chemotherapy and surgery when all costs across the life cycle of patients are considered [4], making it more likely that demand for RT will keep growing over the coming years. In RT, timeliness is crucial and literature shows that delays in the start of treatment increase the risk of local recurrence and tumor progression [5]. In both breast cancer [6] and radical cervix cancer [7], longer radiotherapy waiting times were found to be associated with diminished survival outcomes, and previous research has shown that delay in initiation of radiotherapy may be associated with a clinically important deterioration in local control rates [8]. Besides, unavailability of medical staff was pointed out as one of the main causes for patient dissatisfaction regarding pain management [9]. In RT resources are expensive and limited in capacity, and treatments are prepared and delivered by a multidisciplinary group of specialists with multiple functions and restricted time availability [10]. In addition to variable patient inflows, medical and technological progress makes treatments more and more specialized. Therefore, resource planning and control in RT are complex and time-consuming activities. In this context, advanced analytical models from fields such as systems engineering or applied mathematics have been proposed to help managers of RT centers make better decisions. A recent report published by the Institute of Medicine claims that using systems engineering, timeliness and patient-centeredness in healthcare delivery can be significantly increased [11]. This paper reviews the extent to which operations research techniques have been used to support decision-making in RT, evaluates their (potential) added value and draws lines for future research.

### Operations research and healthcare

Operations research (OR)^1^ is a discipline that combines knowledge from fields such as applied mathematics, computer science, and systems engineering. It encompasses a wide range of techniques for improved decision-making, commonly for real-world problems [12]. Originally, OR emerged as a way to improve military material production during the second world war but methods have continuously grown to model and solve problems in business and industry since then.

During the last decades, a wide range of problems have been addressed to support strategic decision making, facilitate day-to-day hospital management, and solve medical problems related to the healthcare practice [13]. Among the existing OR applications for hospital management and logistics optimization, well-known problems include appointment scheduling [14], staff rostering [15] and operating room planning and scheduling [16]. Given the growing acceptance of OR models to solve problems in healthcare, research on modeling emerging problems receives increased attention, and both a taxonomy for resource capacity planning and control decisions in healthcare and algorithms to solve the most relevant ones have been proposed [17].

### The radiotherapy treatment chain of operations

**Fig. 1:**
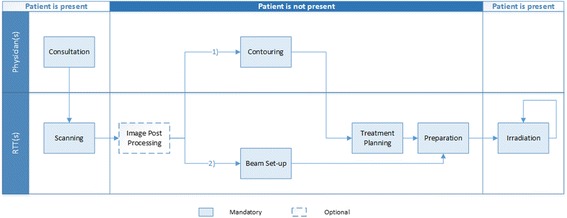
Deployment flowchart of the RT chain

The RT treatment chain is characterized by a sequence of operations, which depends on the characteristics of the tumor (such as location, level of advancement, etc.). Figure 1 depicts a deployment flowchart of the operations involved in external-beam RT. After referral, patients have a consultation with a radiation oncologist, who prescribes one or more diagnostic examinations, such as a computer tomography (CT) scan, a magnetic resonance imaging (MRI) exam, or a positron emission tomography-computer tomography (PET-CT) scan. Thereafter, in most cases the target area is contoured, and the delineation of organs-at-risk takes place in a digital planning system. Once the treatment plan is completed and approved, it is transferred to a linear accelerator (linac) before the first irradiation session. In some other cases, a “beam set-up” is done instead. Here, a skilled RTT defines the angles and intensities of the beams to be irradiated in a certain location, similarly to treatment planning. After a specified number of irradiation sessions, a follow-up period takes place. Although in most types of external-beam RT irradiation sessions can be delivered by a single machine working independently, in other types, such as proton therapy, delivery rooms have a more complicated logistics structure that is not captured by the deployment flowchart of Fig. 1.

The flow of both patients and information is usually influenced by medical and technological constraints. Medical constraints arise when RT is dependent on other forms of treatment such as chemotherapy and/or surgery. In such cases, a time constraint that encompasses a planned delay in the start of treatment emerges. An example is when a patient has surgery before RT and radiation can only be delivered when the wound has healed. Or when a patient receives chemotherapy and a time window for radiation must be followed to ensure the effectiveness of the combined treatment. Technological constraints might occur when only some radiation therapy technologists (RTTs) are trained to carry out a novel treatment or when only a subset of the available linear accelerators (LINACs) is technically capable of delivering RT to a particular cancer type. Moreover, as shown in Fig. 1, staff members (radiation oncologists, RTTs, etc.) are responsible for performing several operations throughout the RT chain, raising the question of how much of their available time should be allocated to each of these operations. In addition, other appointments (e.g. dentist, dietitian) that depend on the availability of the corresponding professionals and can only be undertaken during certain time slots may be needed before the scanning stage, implying increased waiting times for some patients’ throughput. Besides, RT is subject to a considerable number of uncertainties. Daily inflow of new patients, duration of treatment planning activities, and a large number of variables affecting individual care pathways throughout the RT chain appear to be the most significant. Due to this complex logistic environment, the relation between supply and demand in different steps of the chain is not straightforward, and factors limiting the performance of the system - “bottlenecks”—may not always be easy to find. All these factors make the delivery of RT a process with particular characteristics, which brings the need for the development of ‘ad hoc’ approaches to support recurrent decision-making. Nevertheless, knowledge from the OR community can provide the starting point to optimizing RT logistics through the development of innovative, but yet effective decision support systems [18].

### Research aims

There is a wide range of OR applications to solve problems related to medical physics in radiation oncology. A popular example is the design of fluence maps in intensity modulated radiotherapy, i.e. find a fluence pattern over a collection of angles that minimizes the deviation from the desired dose. These applications are discussed by Ehrgott and Holder in [19], but in their review as few as 3 papers covering the logistics aspect of RT treatments are cited. Kapamara *et al.* [20] showed that patient scheduling in RT can be seen as a special case of job-shop scheduling. However, their paper focuses on methods for solving job-shop problems rather than reviewing the application of OR to the RT delivery process.

Although OR methods have been extensively applied to solve problems in RT, literature reviews focusing on resource planning problems are scarce, despite the practical relevance of these problems. To fill this gap, in this paper we identify, study and classify OR models that aim to support managerial decision-making in RT. To that end, the research aims of this study are defined as follows:

1. Identify research papers that cover managerial problems in RT using OR methodologies with at least some empirical material.
2. Position the literature by classifying the studies based on several factors such as the subject of research, the hierarchical nature of decision making and the OR technique(s) employed.
3. Examine the maturity level of implementation of the models and the (potential) impact they have created in practice.
4. Identify the shortcomings in the current literature and provide guidelines for future research.

## Methods

### Scope

Radiotherapy encompasses a wide range of problem types that can benefit from the OR knowledge. According to the framework proposed by Hans *et al.* [21], managerial decisions can be divided in four areas: medical planning, resource capacity planning, materials planning and financial planning. In this work, we focus on resource capacity planning problems. Our goal is to investigate how resources, staff and patients can be efficiently coordinated to optimize objectives such as the minimization of waiting times, or the maximization of capacity use. Therefore, medical or financial problems are excluded from the scope of this study. On the other hand, we focus on OR methods that quantitatively model those problems with measurable performance indicators. While the spectrum of OR methods is wide and not always consistent amongst researchers [22, 23], we classify the methods in six categories: computer simulation, constructive heuristics, metaheuristics, queuing theory, mathematical programming and Markov decision processes. A list of abbreviations and a short description of these methods can be found in Table 1.

**Table 1:** Description of the OR methods

OR method (abbreviation)	Description
Computer simulation (CS)	Process of building an abstract model that mimics the behavior of a real-world or theoretical system, executing the model on a computer and analyzing the output [39].
Constructive heuristics (CH)	Heuristic methods to create and/or improve a candidate solution, step by step, according to a set of rules defined beforehand, which are built based on the specific characteristics of the problem to be solved [40].
Metaheuristics (MH)	General-purpose heuristic algorithms that iteratively improve a candidate solution, designed to solve a wide range of hard optimization problems without having to deeply adapt to the problem at hand [41]. Contrary to CH, MH are problem-independent techniques that can be used as ‘black boxes’. CH and MH are approximation methods, i.e. they do not guarantee that an optimal solution is found. They are used when exact approaches take too much computational time, or when feasibility (or speed) are more important than optimality.
Markov decision processes (MDP)	Mathematical methods to model complex multi-stage decision problems in situations where outcomes are partly random and partly under the control of a decision maker [42].
Mathematical programming (MP)	Optimization methods that aim to mathematically represent a decision problem by defining a set of constraints that bound the values of a set of decision variables, and an objective function to be either minimized or maximized until an optimal solution is found [43].
Queuing theory (QT)	Mathematical methods to model the arrival and departure processes of waiting lines (queues), in order to analyze the congestion and decide the amount of resources required to provide a certain service [44].

### Data sources and search strategy

We performed searches in 6 databases, divided in three categories: medical, technical and multidisciplinary. To find papers within the medical field, we searched EMBASE and PubMed. To look for literature more geared towards engineering approaches, we searched EBSCO Business Search Elite (BSE). In addition, we carried out searches in two multi-disciplinary databases: Web of Science and Scopus. Besides, a search was performed in ORchestra [24], a database created and maintained by the Center for Healthcare Operations Improvement and Research (CHOIR) containing references from the fields of OR and healthcare categorized by medical and mathematical subject. The full strategy and search terms are provided in Additional file 1. As a means to achieve relevant publications not covered by the chosen databases we also checked the references list of the selected papers for snowballing.

### Inclusion/exclusion criteria and paper selection method

Inclusion and exclusion criteria are presented in Table 2. In the aforementioned database search we restricted the search to journal/conference papers and book chapters, and limited the results to papers written in the English language. Besides, due to the fast evolution of both information technologies and algorithms for decision support, we consider that literature studies published before the year 2000 are not likely to be relevant for the purpose of this work. The literature search resulted in 163 different abstracts, from a total of 301 results. Two authors participated in the selection of papers according to the remaining inclusion/exclusion criteria presented in Table 2. We decided to neglect papers focusing on macro-planning, i.e., papers proposing analytical models that support decision making for large scale planning, e.g. involving several RT centers at a regional or national level. Instead, this review focuses on models that aim to solving managerial problems of a single RT center.

**Table 2:** Inclusion and exclusion criteria

Inclusion criteria	Exclusion criteria
Journal paper, conference paper or book chapter	Paper published before 2000
Paper uses an OR method or technique	Paper written in other languages than English
Paper addresses one or more logistics problem in RT	Paper tackles a medical problem
	Paper focus on macro-planning
	Abstract not available online

**Fig. 2:**
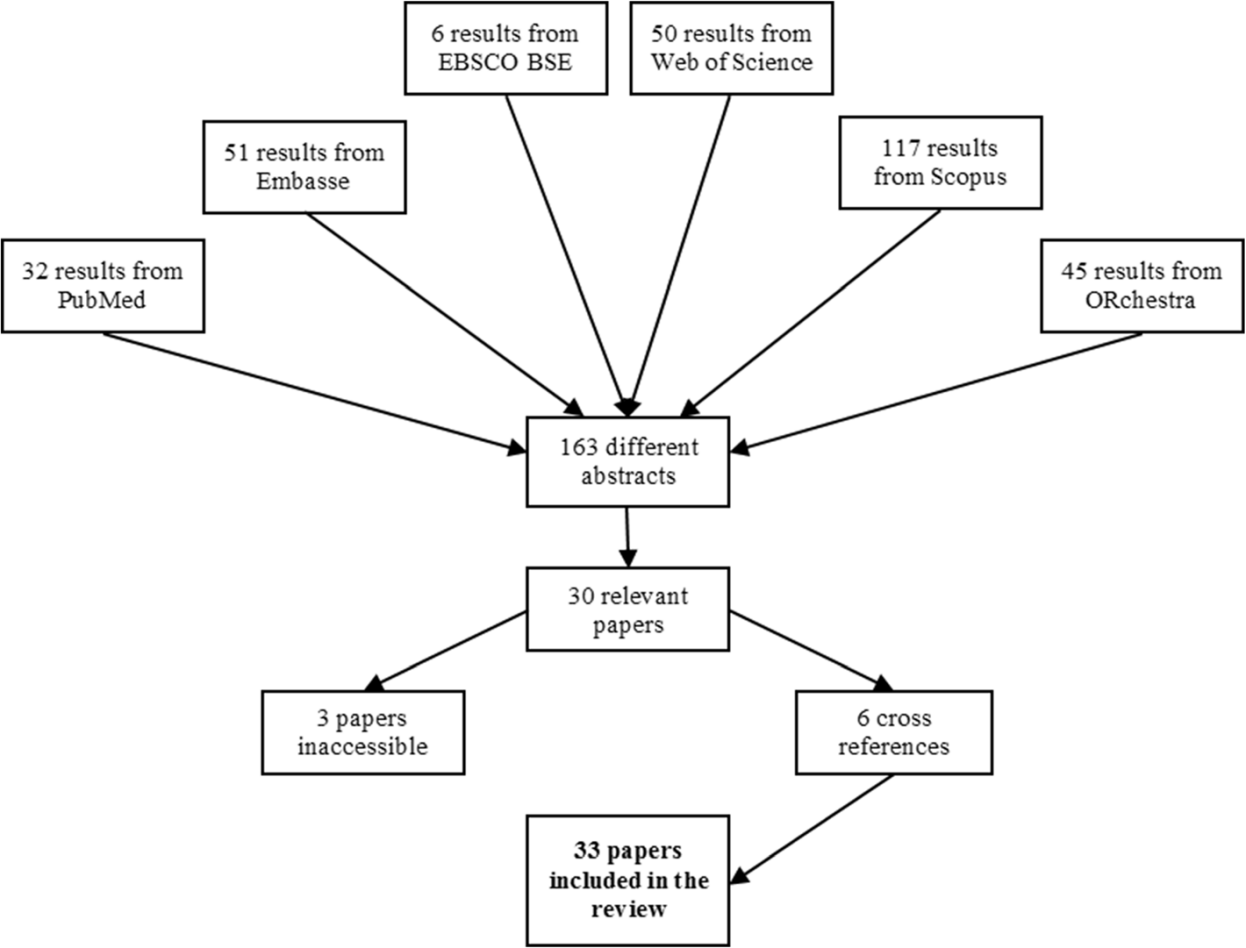
Overview of the selection process

The first author read the title and abstract of all the 163 papers and selected 30 relevant papers. Thereafter, the fifth author read the title and abstract of a random sample of 25% of the 163 papers (41). The matching rate between the authors was 98% (40 in 41), thus the selection procedure undertaken by the first author was considered valid. We were able to obtain, online, the full text of all papers but 3. These 3 papers were submitted to conference proceedings that we were not able to track. The cross reference checks of the remaining 27 papers resulted in 6 additional papers. Therefore, a total of 33 papers were included in this review. Figure 2 depicts an overview of the selection process.

### Data extraction

**Fig. 3:**
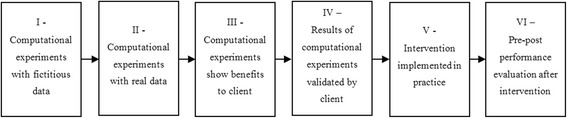
Phases for assessing the extent of implementation

For each paper included in the review we extracted the following information: 1) Subject of research; 2) Hierarchical level; 3) OR method(s); 4) Extent of implementation and 5) (Potential) impact on performance. The subject of research states the type of intervention expected to be taken in practice by the proposed study. It may refer to the problem(s) verified in practice that may have caused the need for a research study, for example. The hierarchical (or organizational) structure was defined in four levels [21]: strategic, tactical, operational offline and operational online. To evaluate the extent of implementation of the models proposed in the literature, we further apply a six stage maturity model as seen in Fig. 3. The maturity model includes the stages through which OR models typically undergo from the end of the development phase to the observation of practical operations improvement.

### Categorization of results

Managerial decisions for planning and control in RT may vary in purpose, scope or objectives, and may be oriented to the long-term, mid-term or short-term operation. We grouped our findings in four sections according to the structure of the decision problems being tackled: 1) Strategic managerial decision making; 2) Resource capacity planning; 3) Patient prioritization; 4) Scheduling.

Strategic managerial decision making refers to finding the best policies that enhance the long-term operation of an RT center. These decisions are commonly linked to the organization’s mission and strategic direction, involving problems such as capacity dimensioning, or the definition of the healthcare delivery process. Strategic decisions usually involve capital investment and are therefore made by on top-level positions of the center’s administration. Because there is a high degree of uncertainty at this level, decisions have a long term planning horizon based on highly aggregated (forecasted) information.

Models for resource capacity planning aim to find the best policies to manage the available capacity of existing machines and staff. These usually cover made for a mid-term planning horizon, and involve the combination of forecasted and known information. Decisions on capacity planning guide and restrict the decisions made at lower levels of the center’s hierarchy. This can be achieved, for instance, by efficiently assigning the available time slots of machines to certain patient groups in order to guide the appointment office when booking appointments for patients, or optimizing the throughput time of a specific process (e.g. the time slot duration for a CT scan). At this level there is a limited flexibility for capacity expansion.

Patient prioritization models attempt to maximize the tumor control probability (TCP) by making decisions on the urgency levels assigned to patients undergoing treatment; certain patients require shorter access times than others. This stratification is related to the characteristics of the tumor and risk of metastasis. Thus, a proper patient prioritization results in a maximized level of satisfaction for the overall population of patients in a waiting list, even if some patients have their waiting time extended in detriment of others.

Scheduling models aim to generate scheduling decisions for patients throughout the RT chain. The goal is to make an efficient planning of the machines’ available capacity by organizing patients in such a way that overall access and waiting times are minimized, delays are avoided, and utilization rates of machines are maximized. Contrary to the previous sections, scheduling decisions typically have a short-term planning horizon, aiming to support the execution of the health care delivery process. Although there is a low flexibility on the supply side, at this level the amount of information available is high. The end goal is to balance the workload in such a way that it can be covered by the available capacity. Studies within this section may be oriented towards a specific operation, or integrate scheduling decisions for a part of the chain of operations, such as the pre-treatment stage, i.e. from referral to the first fraction.

## Results

### Strategic managerial decision making

Table 3 shows the 8 papers that fall within the category of strategic managerial decision making. The subject of research varies among the different scientific publications, with throughput optimization problems being studied the most (50%). Because the majority of the papers address problems at the strategic level (7 in 8), computer simulation is the predominant methodology. Potential improvements were reported, such as the combination of computer simulation and queuing theory performed by Joustra *et al.* [25], which has proven to be capable of increasing the percentage of patients complying with waiting time targets from 39% to 92%. With a similar subject of research, Werker *et al.* [26] presented results that could potentially reduce patients’ waiting times in 20%. Results of both studies were accepted by the corresponding clients, implementation was not reported upon.

**Table 3:** Results for strategic managerial decision making

Reference	Subject of research	Hierarchical level	OR method(s)	Extent of implementation	(Potential) Impact on performance
Thomas [45]	LINACs’ capacity dimensioning	Strategic	CS	II	86% patients begin treatment within 10 days for a spare capacity > = 10\%
Proctor *et al.* [46]	Patient flow analysis	Strategic	CS	III	82% of patients begin treatment within 14 days
Kapamara *et al.* [47]	Patient flow analysis	Strategic	CS	II	2% reduction in patients’ waiting times
Werker *et al.* [26]	Throughput optimization in RT (pre-treatment stage)	Strategic	CS	IV	20% reduction in patients’ waiting times
Joustra *et al.* [25]	Throughput optimization in RT	Strategic	CS + QT	IV	Percentage of patients treated within 21 days increase from 39% to 92%
Aitkenhead *et al.* [48]	Throughput optimization in a proton therapy facility	Tactical	CS	III	Deliver over 100 fractions per working day with 4 delivery rooms
Shtiliyanov *et al.*[49]	Evaluation of radiotherapy centers	Strategic	MP + CS	III	Not mentioned
Price and Wasil [50]	Throughput optimization in a proton therapy facility	Strategic	CS	II	Average increase of 2.1 patients treated per hour

### Resource capacity planning

Five papers tackling resource capacity planning problems were found (see Table 4). Results show that queuing theory and mathematical programming techniques may be very useful to find appropriate solutions within a reasonable time. By efficiently planning of the capacity of treatment machines using these techniques, Li *et al.* [27] were able to reduce the number of weekly time slots needed by 12%. At the tactical level, Bikker *et al.* [28] developed a mixed-integer programming model to allocate the doctors’ capacity for consultation and contouring tasks, as a function of the workload predicted for a mid-term planning horizon. The authors showed a potential access times’ reduction of 15% for regular patients and 16% for subacute patients. These results have been validated by a University Medical Center, and the model is under consideration for implementation. No other implementation reports were found.

**Table 4:** Results for resource capacity planning

Reference	Subject of research	Hierarchical level	OR method(s)	Extent of implementation	(Potential) Impact on performance
Ogulata *et al.* [51]	Capacity planning of a cobalt device	Operational offline	CE + CS	III	No delays in the start of treatment if slack capacity > = 4 patients per day
Joustra *et al.* [52]	Waiting lists management	Tactical	QT + CS	III	Separate queues require 50% less capacity to achieve targets
Li *et al.* [53]	LINACs’ capacity planning	Tactical	QT + MP	I	Not mentioned
Li *et al.* [27]	LINACs’ capacity allocation	Operational Offline	MP + QT	I	Reduction of number of required weekly time slots from 125 to 110
Bikker *et al.* [28]	Doctors’ capacity allocation	Tactical	MP + CS	IV	Access times reduction of 15% for regular patients and 16% for subacute patients

### Patient prioritization

Two papers for patient prioritization were found (see Table 5). Ebert *et al.* [29] presented a non-linear programming model that applies a utilitarian prioritization for patients being treated with curative intent. Their results demonstrated large gains in TCP for some groups of patients at the expense of small reductions in TCP for other groups. However, the simulations revealed to be computationally unrealistic for direct application in a clinical setting. To tackle this drawback, Ebert *et al.* [30] developed an analytical solution that quickly prioritizes patients on a waiting list under the same circumstances as in [29], but using a Lagrangean Multiplier method [31] that leads to the same solution in a much faster way. However, this research is still in a very early stage.

**Table 5:** Results for patient prioritization

Reference	Subject of research	Hierarchical level	OR method(s)	Extent of implementation	(Potential) Impact on performance
Ebert *et al.* [29]	Patient prioritization	Operational offline	MP	I	55% patients with TCP increase
Ebert *et al.* [30]	Patient prioritization	Operational offline	MP	I	Computational time reduction from 1 h to 1 min

### Scheduling

The literature search returned 18 papers addressing scheduling problems (see Table 6). Because both the degree of flexibility and the level of uncertainty are low, these models fall within the operational level of a center’s hierarchy. Most authors apply mathematical programming techniques (9 in 18), thus achieving (near) optimal solutions. However, (meta)heuristic methods appear as a viable supplement or alternative (8 in 18). Optimizing the overall RT chain using both constructive heuristics and metaheuristics, Petrovic *et al.* [32] achieved considerable reductions in waiting times for palliative (34%) and radical patients (41%). Focusing on the pre-treatment stage, Petrovic *et al.* [33] explored similarities between radiotherapy and job-shop scheduling problems commonly encountered in industrial processes, using genetic algorithms to minimize both the average waiting times and the average delays in the start of treatment. Results showed that these indicators were reduced by 35% and 20%, respectively. From the 18 papers found, 12 (67%) propose models for scheduling patients on LINACs. Sauré *et al.* [34] formulated the problem as a discounted infinite-horizon Markov decision process to identify policies that can better allocate the LINACs’ capacity to reduce waiting times. The percentage of treatments initiated within 10 days, for a clinical data-set provided the British Columbia Cancer Agency increased, on average, from 73% to 96%. In contrast, Legrain *et al.* [35], in collaboration with the Centré Integré de Cancérologie de Laval (CICL), proposed a two-step stochastic algorithm for optimal scheduling in an online fashion. Results of computational experiments undertaken using real data instances provided by the CICL showed an average decrease in the number of patients breaching the standards of 50% for acute patients and 81% for subacute patients. As in the previous sections, none of the papers reported a full implementation of the results, with 56% of the studies performing computational experiments only, either with fictitious or real data.

**Table 6:** Results for scheduling

Reference	Subject of research	Hierarchical level	OR method(s)	Extent of implementation	(Potential) Impact on performance
D. Petrovic *et al.* [33]	Pre-treatment scheduling	Operational offline	MH	III	Reduction of average waiting times and tardiness by 35% and 20%, respectively
Kapamara and D. Petrovic [54]	Radiotherapy scheduling	Operational offline	CH + MH	II	Average waiting times of 1.6, 19.1 and 19.4 days for emergency, palliative and radical patients, respectively
S. Petrovic and Castro [55]	Pre-treatment scheduling	Operational offline	MH	II	Not mentioned
Castro and Petrovic [56]	Pre-treatment scheduling	Operational offline	MP + CH	II	11% of all patients exceed the waiting time targets, in average
D. Petrovic *et al.* [57]	Pre-treatment scheduling	Operational offline	MH	II	Reduction of average waiting times for radical (35 to 21.48 days) and palliative (15 to 13.10) patients
D. Petrovic *et al*. [32]	Radiotherapy scheduling	Operational offline	CH + MH + CS	III	Average waiting times of palliative and radical patients reduced by 34% and 41%, respectively
S. Petrovic *et al*. [58]	Treatment scheduling	Operational offline	CH	II	Decrease in the percentage of late patients of up to 40% for palliative patients and 4% for radical patients
S. Petrovic and Leite-Rocha [59, 60]	Treatment scheduling	Operational offline	CE + MH	I	Average weighted tardiness of 0.935 days
Conforti *et al*. [61, 62]	Treatment scheduling	Operational online	MP	III	Increase of 47% in the number of booked treatment sessions
Conforti [63]	Treatment scheduling	Operational offline	MP	I	LINACs’ utilization rates of 95%, in average
Jacquemin *al*. [64]	Treatment scheduling	Operational offline	MP	I	Admission rate of 25.4 patients per week in a fictitious center with 2 LINACs
Burke *et al*. [65]	Treatment scheduling	Operational offline	MP	V	27% of patients breaching the norms
Jacquemin *et al*. [66]	Treatment scheduling	Operational offline	MP	I	4% increase on the percentage of patients treated
Sauré *et al*. [34]	Treatment scheduling	Operational offline	MDP + MP	III	Increase the average percentage of new patients treated within 10 days, from 73% to 96%
Cares *et al*. [67]	Treatment scheduling	Operational offline	MH	I	Not mentioned
Legrain *et al*. [35]	Treatment scheduling	Operational offline	MP	IV	Decrease on the average number of patients breaching the standards by 50% for acute patients and 82% for subacute patients

## Discussion

We observed that there is a growing trend towards applying OR methods for improved decision making in RT over the last 15 years: one paper was published between 2000 and 2005, 13 papers in 2006–2010 and 19 papers in 2011–2015. A total of 33 papers met the inclusion criteria, covering a wide range of problems at various organizational levels with promising results. As for strategic managerial decision making a total of 8 papers were found. At this level, machines’ capacity dimensioning and throughput optimization are the most studied problems with computer simulation as the preferred technique. The 5 papers on resource capacity planning show that suggestions for potential improvements mainly refer to increasing the flexibility by, e.g. implementing a dynamic way of reserving time slots for different patient types, allowing breaks between fractions, or letting treatments start in any weekday. For this type of problems, finding a good balance between demand and supply is of special importance to ensure timely treatments.

We found that scheduling problems are the most studied, with 18 out of the 33 papers (55%). Mathematical programming and (meta)heuristics are the preferred OR methods for patient booking throughout the whole RT chain of operations. We presume that decision makers prefer to get approximate (not optimal) solutions in less computational time, as solutions need to be implemented in a daily/weekly basis. However, the problem structure is usually too complex for applying mathematical programming techniques, which require a high computational effort. From the 18 papers focusing on scheduling problems, 12 (36% of the total papers) address the problem of scheduling patients on treatment machines. An elegant example of finding a proper balance between the processes’ workload and smooth patient flows is a model that focuses on the scheduling of patients throughout the entire RT chain. To demonstrate that, Petrovic *et al.* [32] achieved impressive reductions in waiting times for palliative (34%) and radical (41%) patients using heuristic algorithms and computer simulation together. We found only two papers integrating scheduling decisions for the overall RT chain. The enormous complexity of the optimization models bringing all these scheduling decisions together might explain the low rate of development of scientific studies within this context.

Table 7 summarizes the extent of implementation of the papers included in the literature review. No paper reported a full implementation and performance evaluation of recommendations or software tools, with only one paper referring to a practical implementation being undertaken at the time of publication. Moreover, only four studies had their results validated by the client. Earlier research also reported low levels of actual implementation [36] but publication bias can also play a role. Although we recognize that the extent of implementation of the (scientific) interventions reviewed in this paper may be higher than those reported in the articles, it is also clear that there are many reasons that hamper the translation of theoretical models into practice. First, there are still major issues in getting OR models accepted by clinicians, even when (potential) benefits of innovations are evident [37]. Another factor concerns the development of software tools to be used in the clinic. We found promising models resulting from “in silico” or desk research and/or modelling whereas the translation of the models into a reliable, user-friendly, and bug-free software tool is not straightforward as this part usually falls outside the OR experts’ background. A joint teamwork between software developers and operations researchers is needed to overcome this issue. Data availability may be another reason for the low implementation rates; 9 papers were tested using fictitious data rather than real data. Thus, both the verification and validation of the results become an issue that hampers the acceptance of the model by managers. Further clinicians and OR researchers have different publishing routes and priorities; the former aim at improving effectiveness and efficiency directly in practice, whereas publishing new theoretical findings or innovative algorithms is often sufficient for the latter. A last very practical reason for limited findings of implementation may be that generating evidence on operations improvement is not common practice in healthcare and many incremental improvements are implemented in rapid improvement cycles or by trial and error.

**Table 7:** Results for the extent of implementation

Extent of implementation	Number of papers
I - Computational experiments with fictitious data	9
II - Computational experiments with real data	8
III - Computational experiments show benefits to client	9
IV - Results of computational experiments validated by client	4
V - Intervention implemented in practice	1
VI - Performance evaluation after intervention	0

Although not within the focus of our study, we verified the topic of facility planning on macro level in an additional search. Decisions on long term capacity need and size of RT centers can be of great influence on cost effective allocation of funds. We could only find one study by Shukla et.al. [4], as referred to in the background section, so it is clear that further research on the application of OR methods in RT macro-planning is very relevant.

### Research limitations

We may have missed relevant papers, possibly due to the fact that it concerns an interdisciplinary field. The fact that we found six papers by snowballing demonstrates this.

Although we recognize that more papers within the defined scope might be publicly available, we decided to exclude non-peer reviewed articles in this review. Firstly because a search strategy for these papers may be hard to design, and secondly because these may lack scientific rigor. Yet, we made no distinction between papers based on other factors such as the journal’s impact factor or the quality of the design and data management in the paper.

Implementation stages were scored according to the reported stages in the papers, and no follow-up investigation are done in this review. This is a laborious exercise and has shown to reveal limited response [36]. It is thus not possible to report on the most actual extent of implementation, but we have no indications that implementation in practice is very different from what we found.

Further, there is no deterministic way to define exactly what constitutes an OR methodology, or what the main results of a complex and detailed research work are. Therefore, the data extraction process may have a bias towards the authors’ perspectives.

Still, we believe that this review provides a good overview of the application of scientific knowledge from OR, applied mathematics and systems engineering to operations improvement in RT.

### Future research

Although the range of OR applications in RT is broad and promising results have been reported and some achieved, there is room for future improvement in many directions. Due to new scientific findings related to cancer treatment and technological progress, treatments are getting more specialized and the number of possible care pathways is constantly increasing. This issue creates the need for research in clustering care plans based on the similarities encountered on the corresponding care pathways. Moreover, new devices for improved imaging (such as positron emission tomography–computed tomography) or enhanced radiation delivering (such as the magnetic resonance-LINAC) have been developed. These machines have their own features and limitations, raising the need for new capacity allocation models, as well as the adaption of current models to these new devices. Besides, optimization models should be tested for several real-world data instances in order to strengthen the evidence found by the scientific approaches and ensure the generalization of the models to many different RT centers.

This research produced only one paper proposing a model for scheduling patients in an operational online manner. An investigation area could be the development of innovative models to book patients’ sessions on-site immediately after referral or during consultation. These approaches usually involve the use of stochastic programming methods to find good solutions in the presence of the patient, integrating his/her preferences [38].

Another line for further research is the development of more thorough maturity models to assess the extent of implementation, and identify the main causes for the low implementation rates of OR studies in the healthcare field. Due to the assumptions and simplifications of reality usually done in scientific approaches, it would be interesting to see how the implemented solutions perform in comparison with the theoretical findings. The real extent of implementation could be surveyed by approaching the original authors; earlier experience showed however that this requires creativity and perseverance as organizations and staff positions change frequently, and research is published years after the actual projects took place.

## Conclusions

We show that the literature on OR applications in RT covers a wide range of problems, and considerable benefits can be achieved in terms of both waiting times and resource utilization. But there are still major lines for further research, such as the improved coordination of imaging tests, or the development of online models that enable on-site scheduling of patients immediately upon arrival. With respect to the daily flow of patients, results indicate that scientists and managers tend to believe that bottlenecks are most likely to occur on treatment machines. However, research studies have shown that large gains in waiting times reduction can be achieved if the pre-treatment stage is optimized jointly.

Despite the potential benefits of applying OR methods in RT, implementation rates are still low. We provide suggestions for further development of methods as well as for research priorities.

## Additional files

Additional file 1: Database search strategy. (DOCX 14 kb)

## Abbreviations

CH: Constructive Heuristics
CICL: Centré Integré de Cancérologie de Laval
CS: Computer Simulation
LINAC: Linear Accelerator
MDP: Markov Decision Processes
MH: Metaheuristics
MP: Mathematical Programming
OR: Operations Research
QT: Queuing Theory
RT: Radiotherapy

## Footnotes

1 Sometimes referred to as Management Science.
