# Supplementary material for: Operations research for resource planning and -use in radiotherapy: a literature review
Source: BMC Med Inform Decis Mak. 2016 Nov 25;16:149. doi: 10.1186/s12911-016-0390-4 (PMC5123361; doi:10.1186/s12911-016-0390-4)
Supplement: Additional file 1: — Database search strategy. (DOCX 14 kb) [file 12911_2016_390_MOESM1_ESM.docx]

# Additional file 1: Database search method

Three blocks of search terms were combined with ’AND’, where each block contains a combination of terms with ’OR’. The first block focus on terms that permit to filter research work undertaken specifically in radiotherapy, and is composed by: “radiotherapy”, “radiation oncology” and “radiation therapy”. The second block includes terms related to target optimization problems, filtering documents tackling the specific managerial/logistics problems encountered in RT: “roster(ing)”, “patient (in)flow”, “throughput time(s)”, “wait(ing) time(s)”, “access time(s)”, “process time(s)” “appointment scheduling”, “capacity planning”, “resource allocation”, “patient scheduling”, “patient throughput”, “patient prioritization”, “resource planning”, “workforce”, “capacity dimensioning”, “block planning” and “waiting list(s)”. Finally, the third block of search terms is designed to target OR-based approaches developed to tackle the aforementioned problems: “decision-making”, “decision support tool(s)”, “systems engineering”, “operations research”, “computer model(ing)”, “simulation”, “genetic algorithm(s)”, “evolutionary”, “heuristic(s)”, “constructive approach(es)”, “metaheuristic(s)”, “local search”, “mathematical model(ing)”, “mathematical optimization”, “mathematical programming”, “linear programming”, “integer programming”, “dynamic programming”, “constraint programming”, “queuing”, “queueing”, “markov process(es)”, “multi-objective”, “multi-criteria”, “multiple criteria” and “monte carlo”. Additionally, we used two terms to filter papers tackling two widely studied medical problems, by using the logic gate ’NOT’. Therefore, papers with the term “androgen suppression” or “intensity-modulated” in their title or keywords did not appear in the results retrieved by each database. Additionally, we used medical subject headings (MeSH) terms for the search on PubMed and EMBASSE. In the block of problem-oriented terms we added: “Personnel Staffing and Scheduling” and “Resource Allocation”. As for the third block, we added: “Decision Making”, “Decision Support Techniques”, “Decision Support Systems, Clinical” and “Computer Simulation”. In summary, any research work, in any of the journals covered by the six databases, containing one or more search (or MeSH) terms of each block in its title/abstract/keywords section was found.
